# Neutrophil extracellular traps−related signature predicts the prognosis and immune infiltration in gastric cancer

**DOI:** 10.3389/fmed.2023.1174764

**Published:** 2023-08-10

**Authors:** Mingzhe Li, Zidan Zhao, Tsz Kin Mak, Xiaoqun Wang, Jingyao Chen, Hui Ren, Zhiwei Yu, Changhua Zhang

**Affiliations:** ^1^Digestive Diseases Center, The Seventh Affiliated Hospital of Sun Yat-sen University, Shenzhen, China; ^2^Department of Colorectal Surgery, Harbin Medical University Cancer Hospital, Harbin, China; ^3^Guangdong Provincial Key Laboratory of Digestive Cancer Research, The Seventh Affiliated Hospital of Sun Yat-sen University, Shenzhen, Guangdong, China

**Keywords:** neutrophil extracellular traps, NETs-related signature, immune therapy, NETs-related signature predicts the prognosis, gastric cancer

## Abstract

**Introduction:**

Gastric cancer (GC) is the fifth most prevalent cancer globally, with the third highest case fatality rate. Neutrophil extracellular traps (NETs) are a reticulated structure of DNA, histones, and antimicrobial peptides produced by active neutrophils that trap pathogens. Even though NETs are associated with poorer recurrence-free survival (RFS) and overall survival (OS), the specifics of this interaction between NETs and cancer cells are yet unknown.

**Methods:**

The keywords “neutrophil extracellular traps and gastric cancer” were used in the GEO database for retrieval, and the GSE188741 dataset was selected to obtain the NETs-related gene. 27 NETs-related genes were screened by univariate Cox regression analysis (*p* < 0.05). 27 NETs-related genes were employed to identify and categorize NETs-subgroups of GC patients under the Consensus clustering analysis. 808 GC patients in TCGA-STAD combined with GES84437 were randomly divided into a training group (*n* = 403) and a test group (*n* = 403) at a ratio of 1:1 to validate the NETs-related signature.

**Results:**

Based on Multivariate Cox regression and LASSO regression analysis to develop a NETs-related prognosis model. We developed a very specific nomogram to improve the NETs-clinical score’s usefulness. Similarly, we also performed a great result in pan-cancer study with NETs-score. Low NETs scores were linked to higher MSI-H (microsatellite instability-high), mutation load, and immune activity. The cancer stem cell (CSC) index and chemotherapeutic treatment sensitivity were also connected to the NET score. Our comprehensive analysis of NETs in GC suggests that NETs have a role in the tumor microenvironment, clinicopathological features, and prognosis.

**Discussion:**

The NETs-score risk model provides a basis for better prognosis and therapy outcomes in GC patients.

## Introduction

Gastric cancer (GC) is the fifth most prevalent cancer globally, with the third highest case fatality rate (1). Surgical excision, radiotherapy, and chemotherapy are still the primary treatments for GC, but the prognosis is still unsatisfactory (2). Immunotherapy has been a prevalent topic in the comprehensive treatment of tumors as immunology has advanced. Due to the complex pathogenesis of GC, the tumor microenvironment (TME) composed of fibroblasts, endothelial cells, neutrophils, macrophages, and T/B lymphocytes leads to the heterogeneity of GC patients (3). Increasing research suggests that changes in the composition of resident cell types in the TME may be relevant to immune responses and chemotherapeutic benefits (4). Infiltration levels of CD8 T cells, CD4 T cells, macrophages, and cancer-related fibroblasts (CAF) in TME are associated with the prognosis of numerous cancers, such as gastric cancer, melanoma, bladder cancer, lung cancer, and breast cancer (5–9). Recent studies indicate that tumor-associated neutrophils (TANs) have been essential in carcinogenesis (10). According to cytokine production patterns and effector functions, TANs can polarity into anti-tumorigenic “N1” and pro-tumorigenic “N2” phenotypes. By secreting pro-inflammatory and pro-angiogenic chemokines and cytokines, TANs can facilitate distant metastasis of tumor cells (11, 12).

Neutrophil extracellular traps (NETs) are a reticulated structure of DNA, histones, and antimicrobial peptides produced by active neutrophils that trap pathogens such as bacteria, fungi, and viruses and play a significant role in innate immunity (13). Some literature showed that targeting neutrophil extracellular traps can be a novel strategy to prevent cancer metastasis (14–16). Even though NETs play an active role in fighting pathogen invasion, recent research indicates that cancer cells promote tumor progression by recruiting TANs and releasing NETs into the tumor microenvironment and circulation (17). NETs were discovered in metastatic Ewings sarcoma tissue (18), implying that NETs are associated with tumor metastasis. The relationship between the NETs release of neutrophils stimulated *in vitro* and poor prognosis was subsequently demonstrated in colorectal and cervical cancer (19, 20). In addition, elevated levels of TANs and NETs were found in metastases from colorectal and breast cancers (21). Preoperative serum MPO-DNA levels (a well-established biomarker for circulating NETs) in patients with liver malignancies were associated with poorer recurrence-free survival (RFS) and overall survival (OS) (22). Increased peripheral blood NETs in GC patients are associated with lymph node metastases, poorer short-term outcomes, and progression-free survival (PFS). They can be an independent prognostic factor for GC patients (23). The migration and invasive capacity of GC cells were enhanced by epithelial-mesenchymal transition (EMT) after using NETs to affect them *in vitro* (24). Another study revealed that NETs in the blood and ascites promote distant metastasis of GC cells *in vivo* by modeling postoperative abdominal infectious complications (AIC) (25). Although the NF-κB and TGF-β signaling pathways are involved in the above-affected GC cell phenotype (26), the molecular mechanisms by which NETs affect GC and other immune cells in TME have not been thoroughly understood.

To further investigate the impact of NETs-related genes on the prognosis and treatment of GC patients, transcriptome, clinicopathological, and OS data of GC were obtained from the Gene Expression Omnibus (GEO) and The Cancer Genome Atlas (TCGA) databases. Furthermore, 353 NET-related genes were identified in the GSE188741. The GC prognostic model based on seven NETs-related differential expression genes (DEGs) was constructed using differential analysis, univariate Cox, Multivariate cox, and most minor absolute shrinkage and selection operator (LASSO) analysis, and the predictive efficacy of the model was validated using internal cohort, external cohort, and consensus clustering analysis. In addition, thorough bioinformatics analyses were conducted to evaluate differences in immune infiltration, chemotherapy, and immunotherapy sensitivity between high and low NETs-score groups. Finally, the expression pattern of these seven NETs-associated DEGs in TME was validated using single-cell sequencing data. This study provides new insights for improving the prognosis of GC patients and further investigating the specific molecular mechanisms of NETs for GC.

## Method

### Data collection

The transcriptome data, somatic mutation, and clinical materials of the normal gastric sample (*n* = 32) and GC sample (*n* = 375) were downloaded from TCGA.^1^ In addition, GSE84437 (GC sample = 433), GSE15459 (GC sample = 192), GSE66229 (GC sample = 300), GSE38749 (GC sample = 15), and GSE26253 (GC sample = 432) with detailed characteristic information and survival duration were obtained from the GEO database.^2^ The IMvigor210 cohort (immunotherapy cohort for bladder cancer) expression data and clinical information were obtained from http://research-pub.gene.com/IMvigor210CoreBiologies/. In addition, GSE126044 (immunotherapy cohort for non-small cell lung cancer), GSE135222 (immunotherapy cohort for non-small cell lung cancer), and GSE179351 (immunotherapy cohort for colon cancer and pancreatic cancer) were obtained from the GEO database. The ccRc cohort (immunotherapy cohort for renal cancer) was obtained from https://doi.org/10.1038/s41591-020-0839-y. We also analyzed the response to immunotherapy in GC patients from the PRJEB25780 cohort. Perl scripts were utilized to extract each GC sample’s transcriptome matrix and clinical information and merged for further analysis. Based on R software 4.1.1, the raw data downloaded above were normalized for subsequent analysis using the “limma” package. The data were batch-corrected using the “sva” package, and the processed data from TCGA-STAD and GES84437 were selected and combined for further analysis.

### Retrieval and mining of NETs-related regulators in GC

The keywords “neutrophil extracellular traps and gastric cancer” were used in the GEO database for retrieval, and the GSE188741 dataset was selected. The GSE188741 dataset included mRNA/miRNA/lncRNA sequencing information for AGS GC cell line samples and AGS treated with NETs isolated from the blood of GC patients (27). We downloaded the expression matrix processed by the author. After deleting seven non-coding RNAs, we obtained 353 differential mRNAs utilizing the “limma” package for differential analysis in R 4.1.1. These DEGs were considered to be NETs-related regulators in GC for subsequent analysis.

### Characteristics of the NETs-related regulators

First, based on the “limma” package in R 4.1.1.1, we performed the differential analysis of 353 NETs-related genes in TCGA-STAD (logFC >1, FDR < 0.05) and screened out 130 differentially expressed NETs-related genes. Then, using the “survival” package, 27 NETs-related genes were screened by univariate Cox regression analysis (*p* < 0.05) and visualized by forest map. Meanwhile, somatic mutation prevalence, the genetic locus, and CNV of 27 NETs-related genes were analyzed. At the same time, we also investigate the interaction between different NETs-related genes.

### Consensus clustering analysis

Consensus clustering in unsupervised learning algorithms is commonly applied in cancer research as a classification method (28). The “ConsensusClusterPlus” package determined the number of clusters and their stability. 27 NETs-related genes were employed to identify and categorize NETs-subgroups of GC patients. Additionally, 1,000 replications were conducted to confirm categorization stability. After that, Uniform Manifold Approximation and Projection (UMAP), Principal Component Analysis (PCA), and t-distributed Stochastic Neighbor Embedding (tSNE) were used to validate the reliability of clustering with the R package “ggplot2.”

To further examine the clinical value of the consensus clustering, we perform the Kaplan–Meier survival analysis in different NETs-clusters using the “survival” package of the R software. Furthermore, we evaluated the correlations among the NET subtypes, clinicopathological characteristics, and prognosis. The clinical characteristics included age, gender, and TN stage. We also downloaded “c2.cp.kegg.v7.4.symbols.gmt,” “c2.cp.kegg.symbols.gmt,” “immune.gmt” and “c5.go.symbols.gmt” from the Molecular Signatures Database (MSigDB) database to carry out gene set variation analysis (GSVA), gene set enrichment analysis (GSEA) and single sample gene set enrichment analysis (ssGSEA) analysis. The “GSVA” R package was used to perform GSVA enrichment analysis and ssGSEA analysis. The “clusterProfiler” R package was used to perform GSEA enrichment analysis.

### Construction and validation of the NETs-related prognostic model

GC patients in TCGA-STAD combined with GES84437 were randomly divided into a training group (*n* = 402) and a test group (*n* = 402) at a ratio of 1:1 to validate the NET-related signature. Based on R 4.1.1, we used the “survival” and “glmnet” packages for Multivariate Cox regression and LASSO regression analysis to develop a NETs-related prognosis model. Then, we calculated each patient’s risk score. The calculation formula is as follows:


$$\mathrm{NETs}-\mathrm{score}=\overset{n}{\underset{i=1}{\sum }}{\mathrm{Coef}}_{\mathrm{NETs}-\mathrm{related genes}}*\exp_{\mathrm{NETs}-\mathrm{related genes}}$$

According to the median NET score, patients were categorized as having a high NET score (more than the median) or a low NET score (below the median). Kaplan–Meier analysis was utilized to compare the survival rates in different cohorts. Furthermore, we compare the correlation between NETs-cluster, NETs-score, and patient survival status using the “gglot2” package. The “pheatmap” package shows the distribution of seven prognostic NET-related genes in all GC patients.

### Establishment and validation of the nomogram

First, the independence of the NETs-related signature for OS was validated further in TCGA-STAD using univariate and multivariate Cox proportional hazards regression (CPHR) analysis. Using the “rms” package in R 4.1.1, a nomogram was constructed according to age, stage, and NET score based on independent prognostic outcomes. In the nomogram system, each factor has a corresponding score; the total score is the sum of the scores for all factors in each sample (29). The nomogram was evaluated using ROC curves for the 1-, 3-, and 5-year survival rates. The calibration curves were used to evaluate the consistency between the predicted and observed OS rates.

### Estimation of the gene mutation and immune landscape

For the gene mutation analysis, the somatic mutation data downloaded from TCGA were analyzed using the “Maftools” package in R 4.1.1 to evaluate the differences between the top 20 high-frequency mutated genes in different NET score groups. In addition, we investigated the differences in tumor mutation burden (TMB), microsatellite stability (MSS), low-frequency microsatellite instability (MSI-L), and high-frequency microsatellite instability (MSI-H) between high and low NETs-score groups, as well as the relationship between NETs-score with TMB and MSI. Using one-class logistic regression (OCLR), the CSC index for each sample was calculated, and then the correlation between the NETs-score and CSC index was evaluated.

For immune infiltration analysis, the algorithm CIBERSORT^3^ can estimate the composition of immune cells in samples based on the gene expression matrix (30). Using this algorithm, we calculated the proportions of 22 types of immune cells in GC patients with TCGA-SATD and GSE84437. Then we compared the heterogeneity in immune cell infiltration into the TME between different NETs-score groups. In addition, we evaluated the correlation between seven prognostic NETs-related genes and these 22 kinds of immune cells. The study also evaluated differences in common immune checkpoints between low and high NET score groups.

### Immunotherapy evaluation based on NETs-score

Previous research has found that malignant tumors can be categorized into six immune subtypes (C1-C6) (31): wound-healing (C1), IFN-γ dominant (C2), inflammatory (C3), lymphocyte-depleted (C4), immunologically quiet (C5), and TGF-β dominant (C6). Another study classified all 419 patients receiving immunotherapy from IMvigor 210 and TCGA into four categories based on cytotoxic lymphocyte (CTL) and lncRNA characteristics (32): immunological activity(TCGA Subtype-I), immune rejection(TCGA Subtype-II), immune dysfunction(TCGA Subtype-III), and immune desert (TCGA Subtype-IV). We evaluated the difference between high and low NET score groups in the TCGA-STAD and IMvigor 210 cohorts for various immune subtypes. Using “RColorBrewer” in R 4.1.1.1, visualize the above results. In addition, we employed immunophenotype scores (IPS) to predict the response to immune checkpoint inhibitors (ICIs) based on the expression of significant components of tumor immunity. Immunogenicity was positively connected with the IPS score (33), calculated using a scale ranging from 0 to 10 and based on the z score of gene expression of representative cell types. The Cancer Immunome Atlas (TCIA, http://tcia.at/home) was used to calculate the IPS for each TCGA-STAD GC patient, and then we compared the differences in IPS across the different NET score groups. Moreover, patients from the IMvigor210 and immunotherapy cohorts were classified using the NET score. We also analyzed the prognostic value of NET scores in different external immunotherapy coorts (KM survival analysis based on the best cut-off of NET score).

For the predicted assessment of the patient with immunotherapy in the prognostic value of NETs-score, the time-dependent ROC curve analysis was performed to obtain the area under the curve (AUC). In addition, we not only downloaded the TIDE score online^4^ but obtained the TIS score by calculating the average value of log2-scale normalized expression in the 18 signature genes. In addition, more relevant immune indicators were used as recent articles (34, 35). After that, we revealed the results for comparing the prognostic between the NETs-score, TIDE, and TIS by using the R package of “timeROC” and performed time-dependent ROC curve analyses to obtain the area under the curve (AUC). The “survival” and “ggplot2” packages were applied to plot OS curves, ROC curves, and various immunotherapy response levels.

### Drug sensitivity analysis

We downloaded the dataset of anti-cancer medications from the Genomics of Drug Sensitivity in Cancer (GDSC) website^5^ and used the “oncoPredict” package in R4.1.1 to predict the chemotherapeutic drug sensitivity of GC patients in TCGA-STAD and GSE84437. Drugs with median IC50 < 1 were screened, which were considered to be powerful drugs for GC treatment. Finally, the sensitivity of these drugs in different NET score groups was statistically tested to evaluate the different response levels of patients with different NET scores.

### Pan-cancer analysis of the NETs-score model

We ranked all TCGA patients with 33 different types of cancer based on the NET score and visualized the distribution of the NET score in different cancers. The correlation between NET score and TME, immune cells, stemness indices, TMB, MSI, and CD274 was further analyzed. In addition, we analysis the correlation of NETs-score with chemokines, chemokines receptors, immune activation regulators, immunosuppressive regulators, and Tumor Inflammation Signature score (TIS score) in pan-cancer.

### Immunohistochemistry staining (IHC) for the signature genes

We compared the expression patterns of signature genes between normal and cancer tissues according to The Human Protein Atlas database (HPA, https://www.proteinatlas.org/, accession date: April 2022).

### Statistical analysis

R (version 4.1.1) was used to perform all statistical analyses and visualizations. The specific R packages used are described in each section. The t-test was used to compare two groups, one-way ANOVA was used to compare multiple groups, Pearson correlation analysis was used to evaluate correlations, and Kaplan–Meier analysis was used to evaluate survival. Any *p* < 0.05 was considered statistically significant.

## Result

### Identification of NETs-related genes in GC

**Figure 1:**
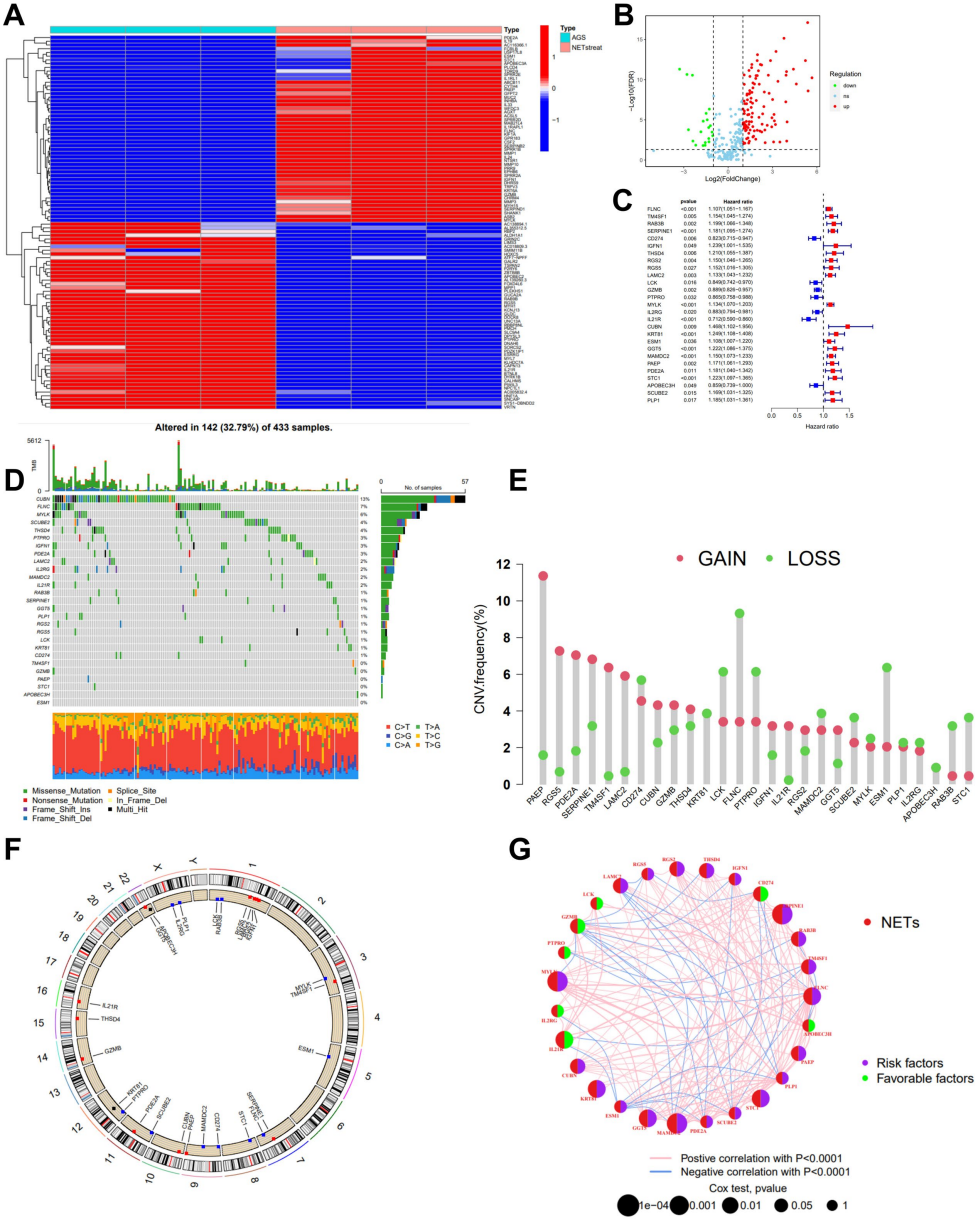
Identification and characteristics of NETs-related regulators in GC. **(A)** Gene expression heat map of NETs-related genes in AGS. **(B)** Volcano plot showing the differentially expressed NETs-related genes in TCGA-STAD. Blue dots represent down-regulated NETs-related genes, and red dots represent up-regulated NETs-related genes. **(C)** The forest plot shows the top 27 NETs-related genes via the univariate Cox regression analysis in TCGA-STAD combined with GSE84437 (*p* < 0.05). **(D)** Mutation frequencies of 27 NETs-related genes in TCGA-STAD. **(E)** Frequencies of CNV gain, loss, and non-CNV among NETs-related genes. **(F)** Locations of CNV alterations in NETs-related genes on 23 chromosomes. **(G)** Interactions among NETs-related genes in GC, and the thickness of lines represent the strength of the association between NETs-related genes. Blue and pink represent negative and positive correlations, respectively.

The process flow chart of our study is shown in Supplementary Figure S1. First, we acquired the data on NET-related gene expression from the GEO database (GSE188741). In the differential expression genes analysis, there were 353 differentially expressed genes retrieved from the GEO cohort, including 212 up-regulated genes and 141 down-regulated genes in the NETs stimulated GC cells line compared with the GC cells line of AGS (Figure 1A). Meanwhile, we intersected these genes with the differential expression genes from the transcriptome profiling data of the Stomach adenocarcinoma (STAD) project from the Cancer Genome Atlas (TCGA) database. The intersecting list yielded 130 differentially expressed NETs-related genes, of which 110 were up-regulated, and 20 were down-regulated in tumor samples as compared to normal samples (Figure 1B). By comparison, these potential genes suggested a function in tumorigenesis that needs to be addressed in future analyses and trials. 27 NETs-related genes from 130 genes in the intersecting list that were highly connected with GC patients were identified by a univariate Cox survival analysis, and Kaplan–Meier analysis was used for the following analysis, shown in Figure 1C and Supplementary Figure S2 (*p* < 0.05).

We examined the gene mutations to understand the types of mutations in GC patients in accordance with the 27 NETs-related genes (Figure 1D). At the genetic level, NET-related regulator mutations were found in 142 of the 433 samples (approximately 32.79%). The analysis showed that most mutations were occurring in CUBN. In addition, missense mutations predominated among the 27 genes connected to the Nets. For 27 NETs-related genes, we calculated the frequency of CNVs and found alterations in 27 NETs-related genes with chromosomal CNVs (Figures 1E,F). PAEP was illustrated as a frequent modification, with most of the changes focusing on copy number amplification on the nine chromosomes. A network was created to show the landscape of the selected genes’ interconnections, regulator linkages, and prognostic significance in patients with GC.

### Identification of NETs subtypes in GC

**Figure 2:**
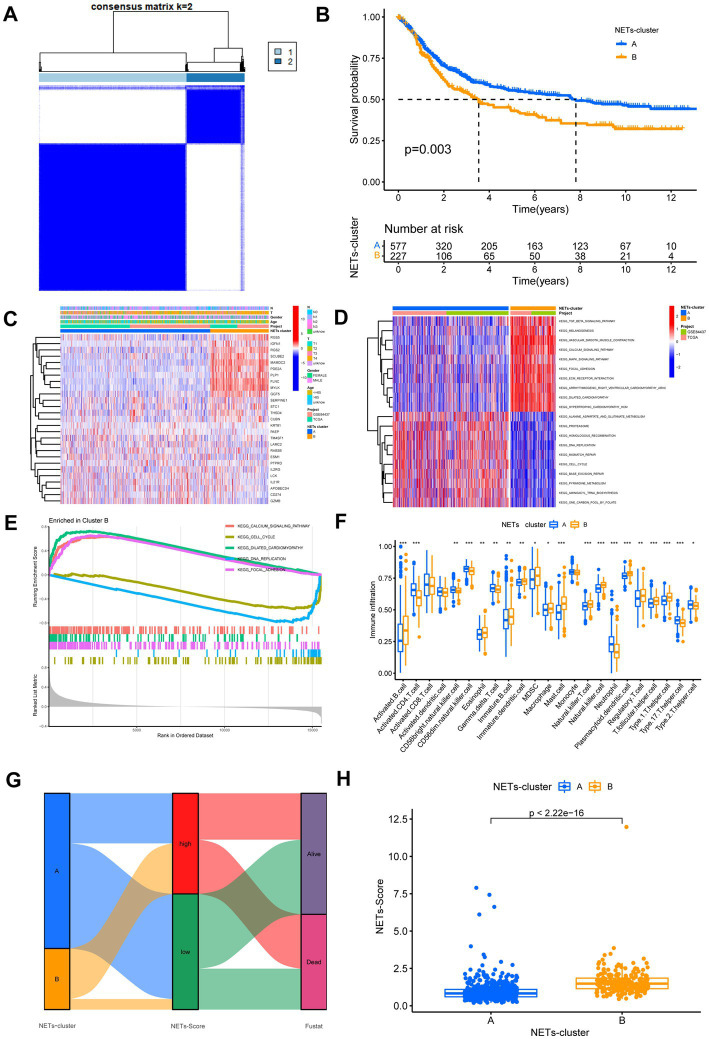
Identification of NETs-subtypes and construction of NETs-related prognosis signature in GC. **(A)** Consensus matrix heatmap defining two clusters (*k* = 2) and their correlation area. **(B)** Overall survival of the two NETs subtypes. **(C)** Differences in clinicopathologic features and expression levels of NETs-related genes between the two NETs subtypes. **(D,E)** GSVA and GSEA analysis focused on the differential enrichment of KEGG pathways between NETs-subtypes A and B. **(F)** The 22 infiltrating immune cell types in the two NETs-subtypes. **(G)** Alluvial diagram of the subtype distributions in groups with different NETs-score and survival outcomes. **(H)** Differences in NETs-score between two NET subtypes. *p < 0.05, **p < 0.01, ***p < 0.001.

First, we used univariate Cox regression and Kaplan–Meier analysis to investigate and identify the selected NETs genes of predictive value in the 808 GC patients. Based on the expression levels of 27 NETs-related regulators, we identified various regulatory patterns using the unsupervised clustering technique. The results showed that *k* = 2 appears to be the optimum option for dividing the entire cohort into subtypes A (*n* = 581) and B (*n* = 227) (Figure 2A). The results of the survival study indicated that Cluster B had a higher survival probability than Cluster A (Figure 2B). Figure 2C showed that the advanced TNM stages, particularly the T stage, were also associated with the NETs-related gene subtype B patterns. Moreover, GSVA enrichment analysis was used to examine the differences in biological behavior between these two patterns (Figure 2D). It revealed that cluster B was enriched for the top five pathways highly related to focal adhesion, dilated cardiomyopathy, cell cycle, DNA replication, and calcium signaling (Figure 2E). We investigated the 22 immune cell kinds infiltrating the two GC subtypes (Figure 2F). The findings indicated that, except for activated CD8 T cells, activated dendritic cells, and monocytes, the most infiltrating immune cell significantly differed between the two GC subtypes. Following that, we confirmed that the 27 NETs-related regulators could be used to discriminate the two regulatory patterns (Supplementary Figure S3). According to this research, the 27 NETs-related regulators might be utilized to distinguish between the two regulatory patterns.

### Establishment of risk assessment model and survival outcomes in GC

DEGs associated with subtypes were used to create the NETs-score. Figure 2G displays the patient distribution across the two NETs subtypes and two NETs-score groups. After LASSO regression analysis, 17 RFS-associated genes were still present according to the least partial likelihood of deviance (Supplementary Figure S4). Seven genes (SERPINE1, LAMC2, MYLK, IL21R, KRT81, MAMDC2, and PAEP) were ultimately retrieved after multivariate Cox regression analysis to create the risk score, known as the “NETs-score.” The multivariate Cox regression analysis’s findings led to the following construction of the NETs-score:


$$\begin{array}{l} \mathrm{Risk score}=\mathrm{expression level of}\;{\mathrm{SERPINE1}}^{*}\left(0.16177\right)\\ \;\;\;\;\;\;\;\;\;\;\;\;\;\;\;\;\;\;\;\;\;\;\;\;\;\;\;\;\;+\mathrm{expression level of}\;{\mathrm{LAMC2}}^{*}\left(0.09126\right)\;\\ \;\;\;\;\;\;\;\;\;\;\;\;\;\;\;\;\;\;\;\;\;\;\;\;\;\;\;\;\;+\mathrm{expression level of}\;{\mathrm{MYLK}}^{*}\left(0.09607\right)\\ \;\;\;\;\;\;\;\;\;\;\;\;\;\;\;\;\;\;\;\;\;\;\;\;\;\;\;\;\;+\mathrm{expression level of}\;\mathrm{IL}2{\mathrm{1R}}^{*}\left(-0.39476\right)\\ \;\;\;\;\;\;\;\;\;\;\;\;\;\;\;\;\;\;\;\;\;\;\;\;\;\;\;\;\;+\mathrm{expression level of}\;\mathrm{KRT}81^{*}\left(0.15615\right)\\ \;\;\;\;\;\;\;\;\;\;\;\;\;\;\;\;\;\;\;\;\;\;\;\;\;\;\;\;\;+\mathrm{expression level of}\;{\mathrm{MAMDC2}}^{*}\left(0.12371\right)\\ \;\;\;\;\;\;\;\;\;\;\;\;\;\;\;\;\;\;\;\;\;\;\;\;\;\;\;\;\;+\mathrm{expression level of}\;{\mathrm{PAEP}}^{*}\left(0.16192\right). \end{array}$$

**Figure 3:**
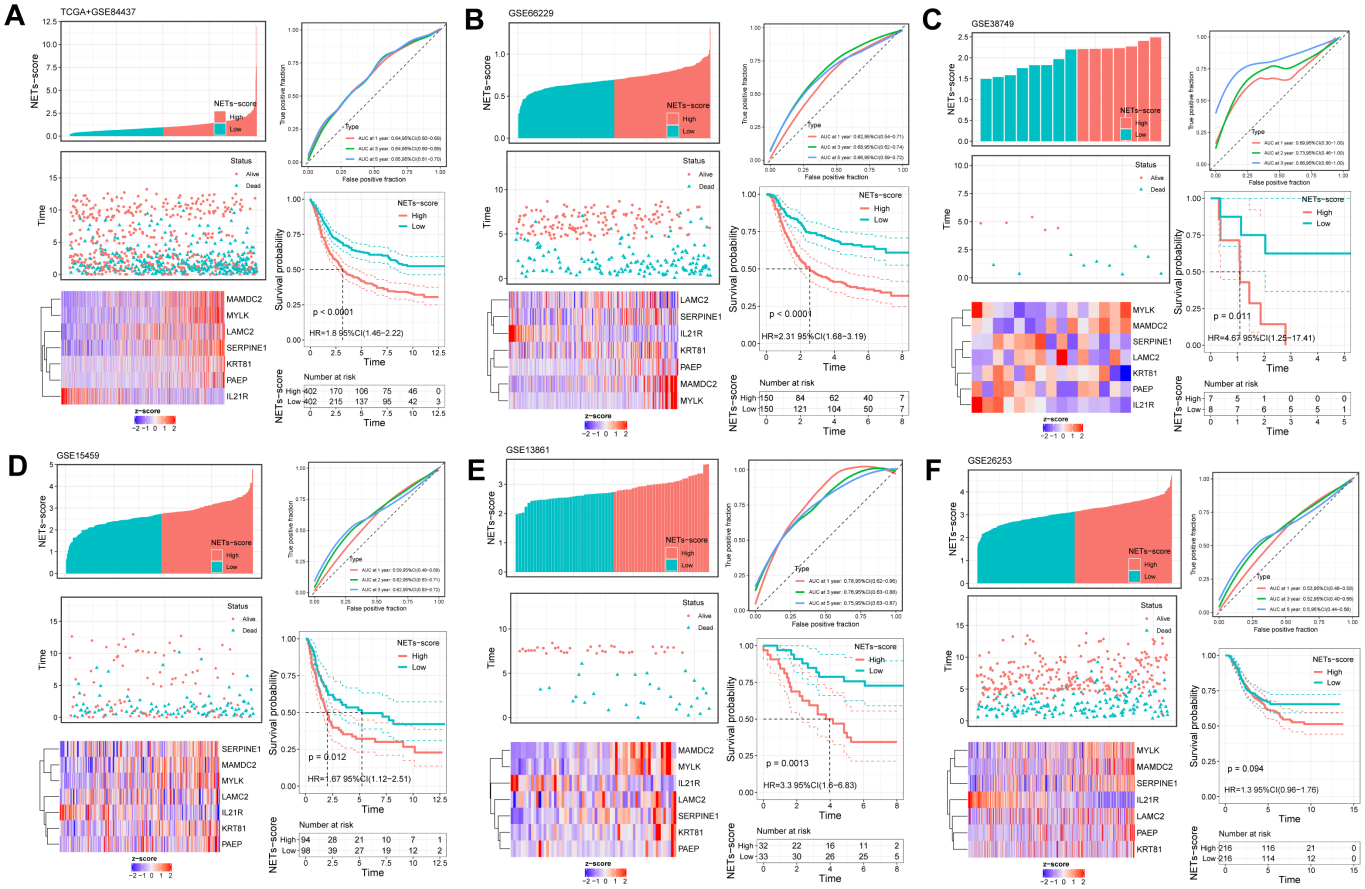
Validation of the prognostic NETs-related signature based on NETs-score (KM survival analysis based on the median NETs-score). The upper left parts are distribution plots for the relationship between NETs-score and survival status; the lower left parts are heatmaps for the seven prognostic NETs-related genes in the cohorts; the upper right parts are ROC curve for the NETs-score in the different internal and external cohorts; the lower right parts are survival curves between high- and low-NETs-score groups. Internal cohorts: **(A)** TCGA combined with GSE84437; External cohorts: **(B)** GSE66229; **(C)** GSE38749; **(D)** GSE15459; **(E)** GSE13861; **(F)** GSE26253.

Further study of the risk score application revealed a substantial variation in the subtypes of NETs (Figure 2H). We investigated the variance in NETs-Score gene expression between the two NETs-score groups (Figure 2H). Figure 3 shows that patients in low-risk categories had better OS than high-risk patients (*p* < 0.05) regardless of the TCGA combined GSE84437 internal cohort, GSE66229 external cohort, GSE15459, GSE13861 or GSE38749. In addition to this study, the Kaplan–Meier analysis of OS was performed using the TCGA combined GSE84437 for internal cohort and external cohort of GSE15459, GSE66229, GSE13861, and GSE38749 (Figure 3). Consistent with Figure 3’s findings, the patient within low-risk groups had a better OS than the high-risk patients (*p* < 0.05), expected GSE26253 (Figure 3F).

### Establishment nomogram and assess the clinicopathologic

**Figure 4:**
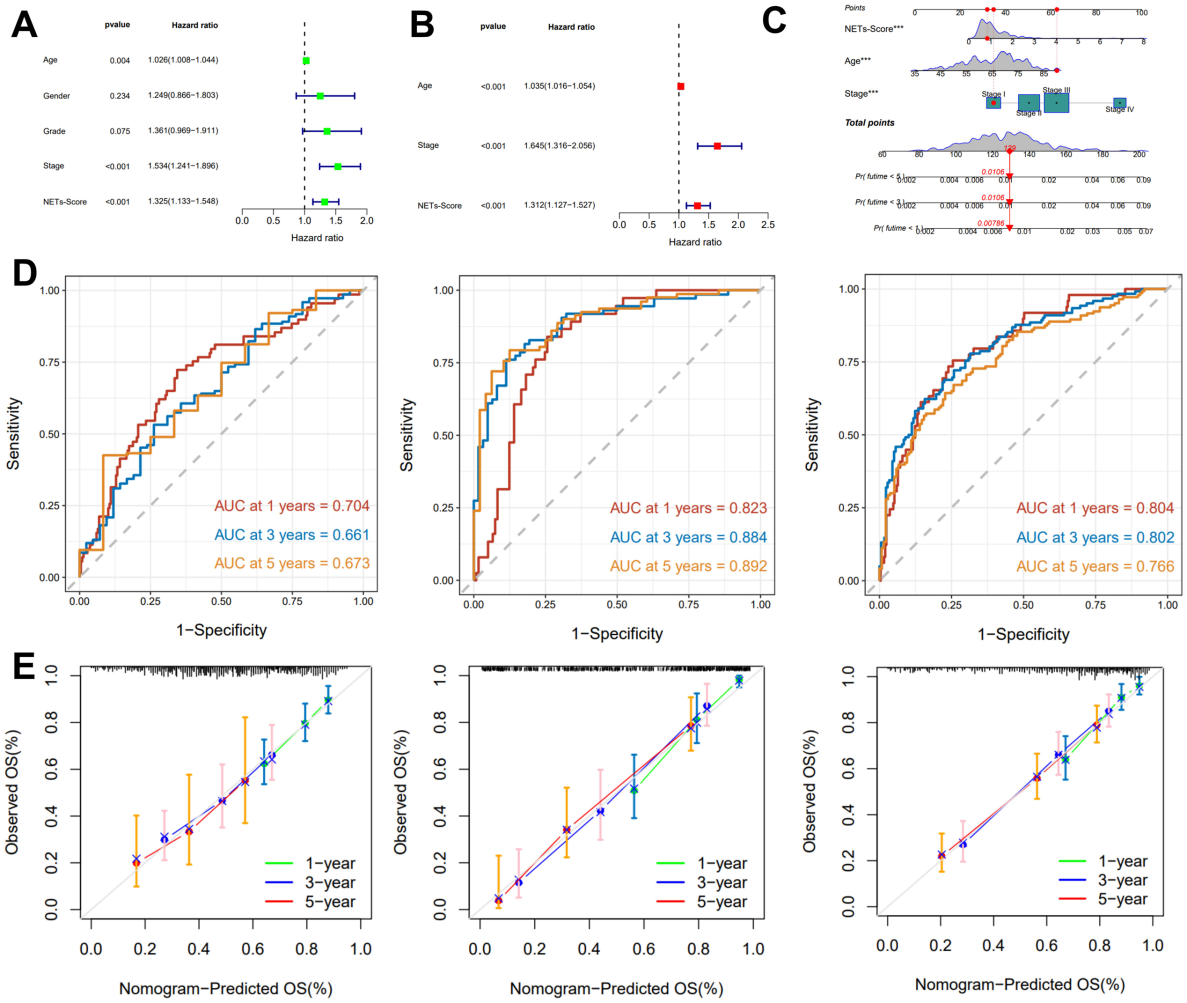
Construction and validation of the nomogram. **(A,B)** The univariate and multivariate Cox regression analyses included different clinicopathological features. **(C)** Nomogram model for predicting the 1-, 3-, and 5-year OS of GC patients. **(D)** The time-dependent ROC curves of the nomogram for 1-, 3-, and 5-years OS in TCGA, GSE15495, and GSE66229 cohorts. **(E)** Calibration curves of the nomogram for predicting 1-, 3-, and 5-year OS in the TCGA, GSE15495, and GSE66229 cohorts.

The finding showed that age, NETs score, and stage were substantially linked with the prognosis of GC under the univariate Cox regression analysis (Figure 4A). After the clinicopathologic characteristics were taken into account in a multivariate Cox regression analysis, the result revealed that the NET score appeared as an independent predictive factor (Figure 4B).

A nomogram including NETs-score and clinicopathological features was created to predict 1-, 3-, and 5-year RFS rates in patients with GC due to the unfavorable clinical usefulness of NETs-score in predicting RFS in patients with GC (Figure 4C). Our AUC analyses on the nomogram model in the TCGA cohort, GSE15459, and GSE66229, showed improving accuracy for RFS at 1, 3, and 5 years (Figure 4D). According to the calibration plots, the suggested nomogram performed similarly to an ideal model in both TCGA cohorts, GSE15459 and GSE66229 (Figure 4E).

### Relationship of TMB, MSI, and CSC index

**Figure 5:**
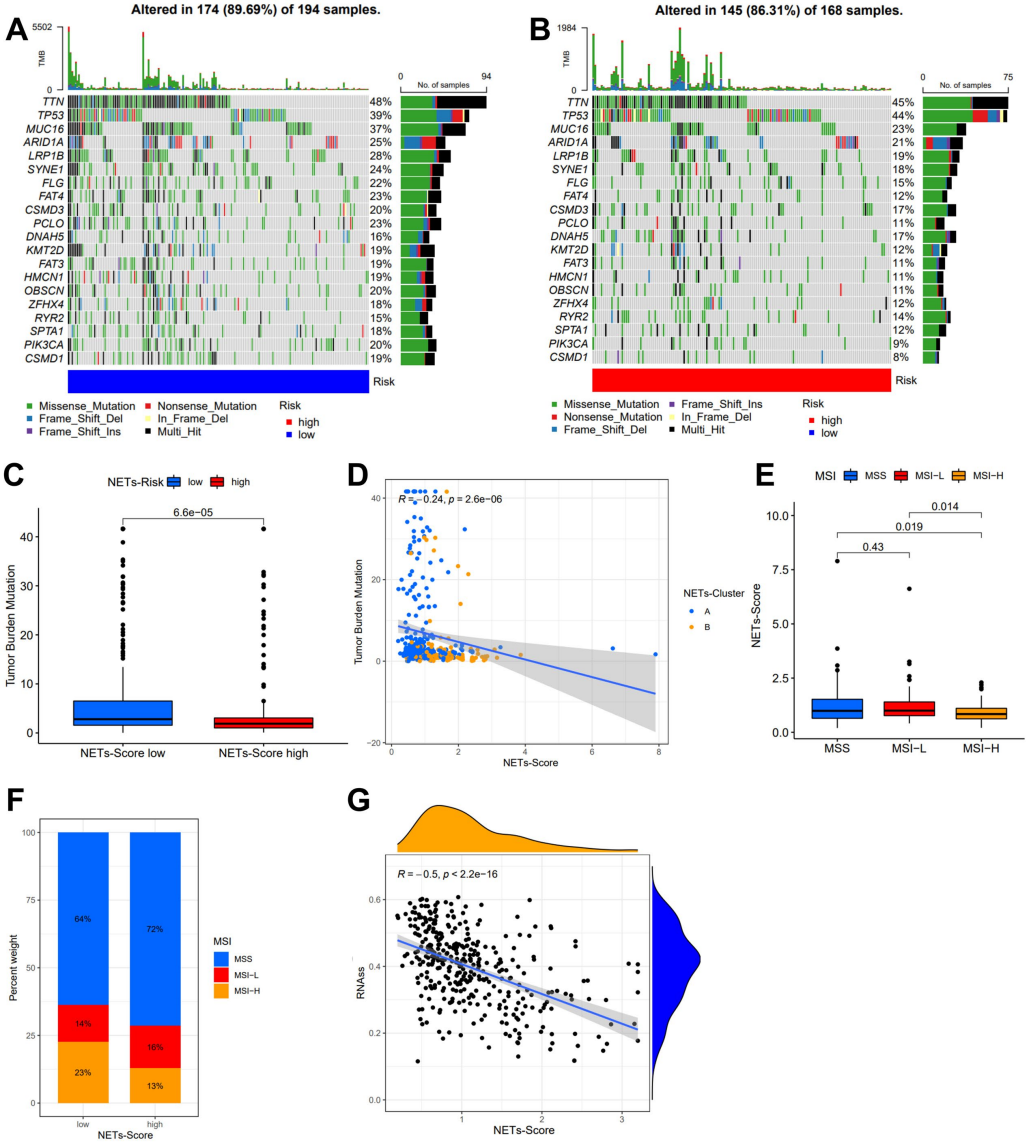
Characteristic in gene mutation and relationship of NETs-score with TMB, MSI, and CSC index. **(A,B)** Significantly mutated genes in the TCGA-STAD samples of the low and the high NETs-score subgroups, respectively. Mutated genes (rows, top 20) are ordered by mutation rate; samples (columns) are arranged to emphasize mutual exclusivity among mutations. The right shows the mutation percentage, and the top shows the overall number of mutations. The color-coding indicates the mutation type. **(C)** TMB in two NETs-score subgroups. **(D)** Relationships between NETs-score and TMB in NETs-subtypes. **(E,F)** Relationships between MSI and NETs-score. **(G)** Relationships between CSC index and NETs-score.

In the low-risk subgroup (Figure 5A) and the high-risk subgroup (Figure 5B), we determined the top 20 genes with the greatest mutation rates. The findings showed that missense mutation and multiple hits were the most frequent mutation types. TTN and TP53 not only had mutation rates of more than 40% in both groups, but these genes were also the most prevalent in both. Additionally, we examined the connection between the risk score and TMB. In comparison to the high-risk grouping, the low-risk subgroup had much greater TMB expression (Figure 5C). Figure 5D further showed a substantial negative correlation between the risk score and TMB in gene subtypes (*r* = −0.24, *p* < 0.05). Furthermore, we found that a high NET score was associated with microsatellite stable (MSS) status, while a low NET score was highly associated with MSI-H status (Figures 5E,F). The risk score was found to have a substantial correlation with the CSC index (*r* = −0.5, *p* < 0.05), as shown in Figure 5G. The results showed that stem cell characteristics were more evident in GC cells with a lower NET score and that there was less cell differentiation.

### Immune infiltration in NETs-score of gastric cancer (GC)

**Figure 6:**
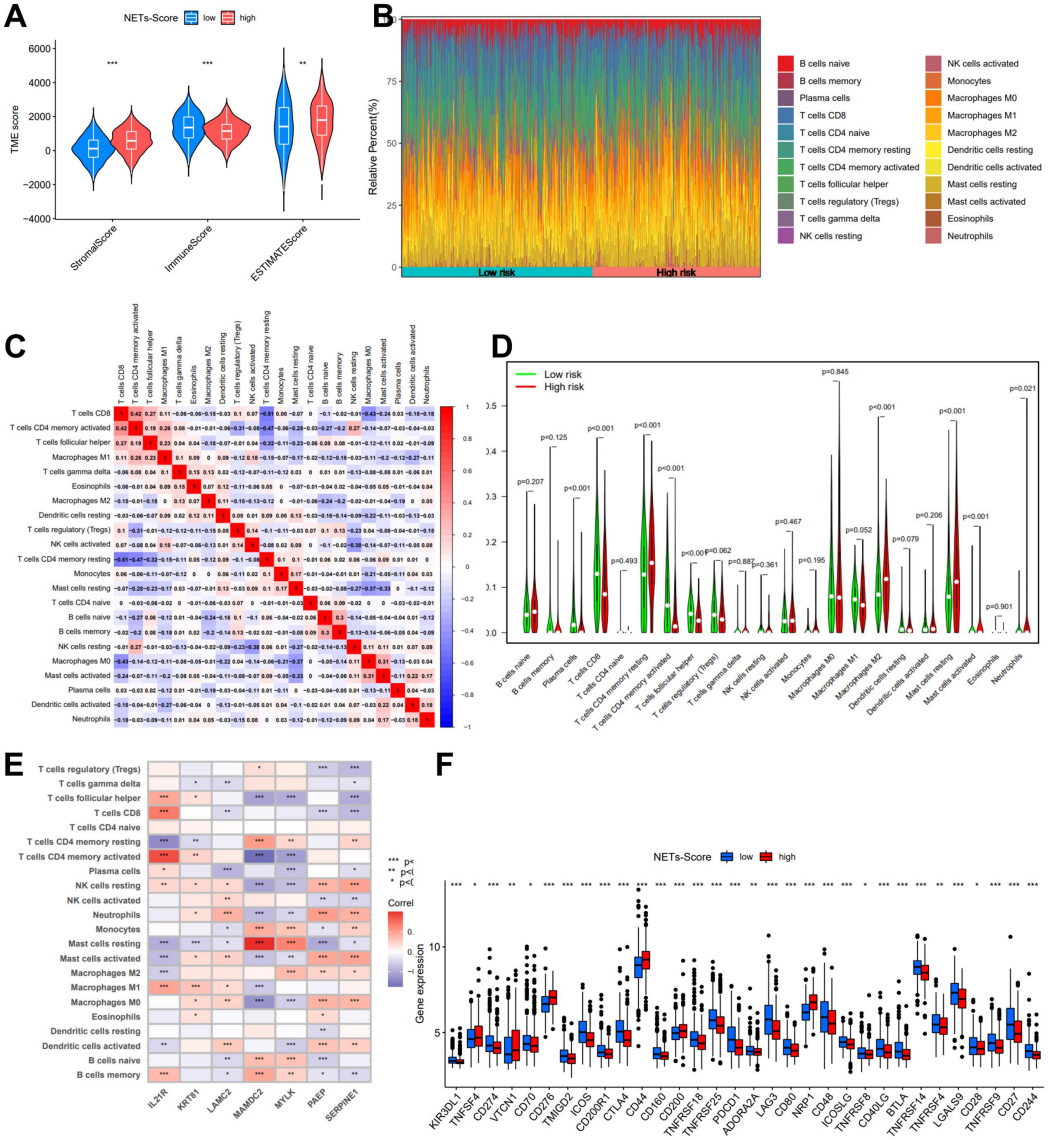
The immune microenvironment of GC at different NETs-score. **(A)** Estimate score of the expression profile in different NETs-score groups. **(B)** Composition of immune cells in different NETs-score groups. **(C)** Correlation between immune cells. **(D)** The relative immune infiltration level of 22 immune cells between different NETs-score groups. **(E)** Correlation between immune cells and seven prognostic NETs-related genes. **(F)** Expression of all immune checkpoint genes in different NETs-score groups. *p < 005, *p < 0.01, ***p < 0.001.

A low NET score was closely connected with a high immunological score, whereas a high PRG score was associated with a high stromal score and estimate score (Figure 6A). To calculate the fractions of 22 immune cells, the gene expression matrix from the TCGA database in GC was uploaded to CIBERSORT online. We then looked at the immune cell makeup in various risk categories in the TCGA database of GC samples (Figure 6B). The results showed that plasma cells, T cells CD4 memory, and T cells follicular helper were more prevalent in the low-risk category, whereas the patients in the high-risk subgroup had substantially greater proportions of neutrophils, macrophages M2, and mast cells (*p* 0.05) (Figures 6C,D).

The relationship between immune cells in patients with GC may offer hints for a better comprehension of the immune microenvironment in particular types of tumors. Based on the TCGA, we discovered that the NETs-score was strongly connected to B cells naïve, Macrophages M2, Mast cells, and T cells CD4 (Supplementary Figure S5). The seven genes were also demonstrated to be significantly connected with the bulk of immune cells (Figure 6E). Finally, we investigated the immune checkpoint gene expression in relation to the various risk score subgroups. The findings demonstrated that the low-risk score group was greater expression of numerous immune checkpoints, such as PD-1 and CTLA-4, as well as the T cell inflamed score (Figure 6F).

### Immunotherapy efficiency

**Figure 7:**
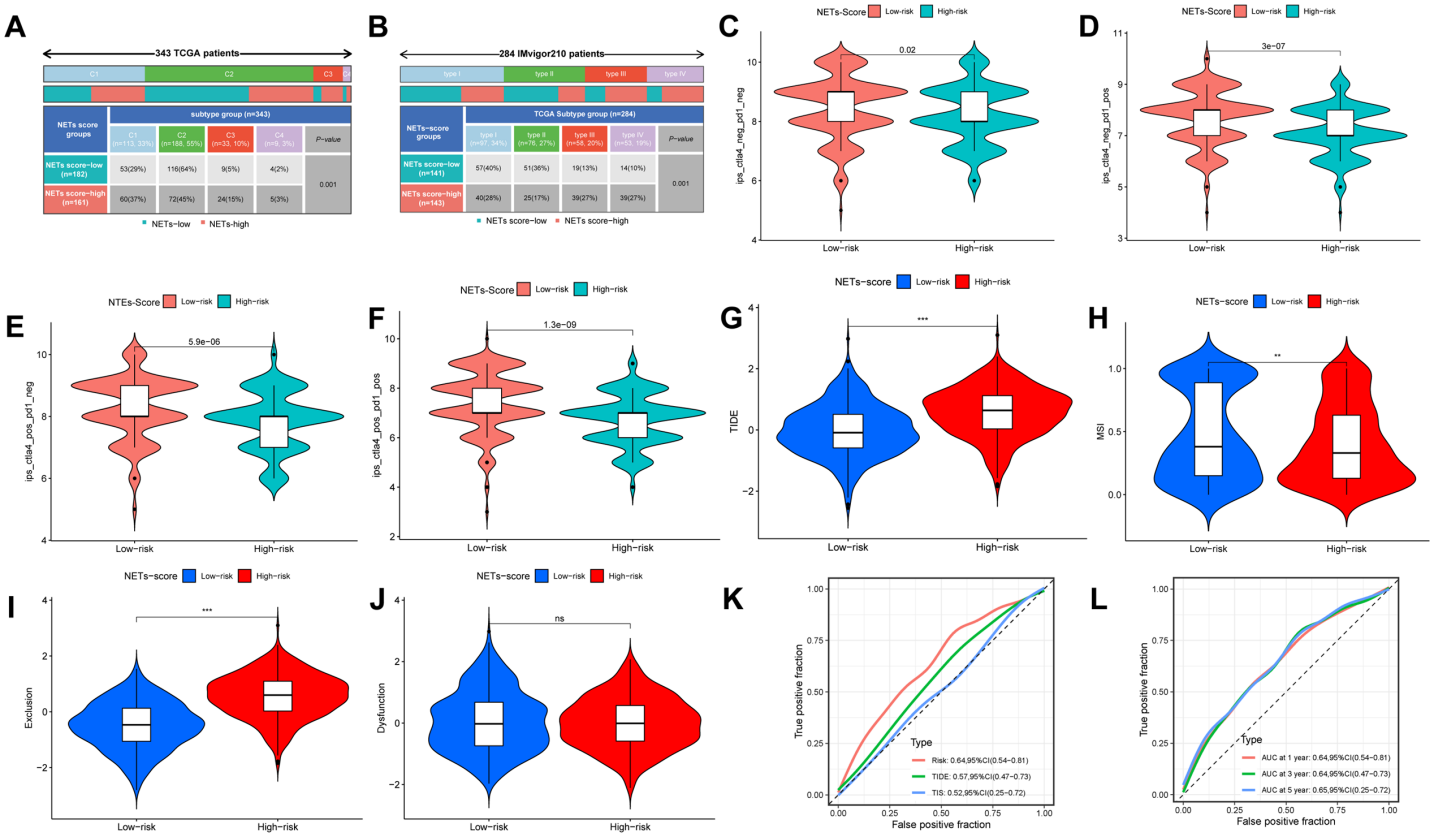
The prognostic value of NETs-score in immunotherapy. **(A)** The immune subtypes were significantly related to the different NETs-score groups in TCGA-STAD. **(B)** The TCGA subtypes were significantly related to the different NETs-score groups in IMvigor210. **(C–F)** The vioplot of the different expressions of CTLA4 and PD-1 between different NETs-score groups (TCGA-STAD). **(G–J)** TIDE, MSI, T cell exclusion, and T cell dysfunction score in two NETs-score subgroups (internal cohort), respectively. **(K,L)** ROC analysis of NETs-score, TIDE, and TIS on OS in the internal cohort. NS: no significant, **p < 0.01, ***p < 0.001.

We discovered that there was a substantial relationship between the immunological subtypes and the risk for the two IRGPI subgroups (Figure 7A). Meanwhile, we revealed the 4 classes in the IMvigor210 trial that there were more type 1 and type 2 in the low-risk subgroup, while more type 3 and type 4 in the high-risk subgroup (Figure 7B, *p* < 0.05). We examined the relationship between risk and immunophenoscore (IPS) in GC patients to forecast the response to immune checkpoint inhibitors in order to evaluate the potential efficacy of immunotherapy under the clinical in various risk groupings (ICIs). Only three immunological checkpoints—cytotoxic T lymphocyte antigen-4 (CTLA-4), programmed cell death protein 1 (PD-1), and programmed death ligand-1—represent the key immune checkpoints for the IPS (PD-L1). Immune checkpoints were therefore employed to assess the potential effectiveness of ICI therapy (Figures 7C–F). As a consequence, we discovered that there were much higher in the low-risk group, which was classified by risk, indicating that the low-risk group had greater immunogenicity on ICIs. These findings collectively showed that the low-risk group had a higher likelihood of mounting an immunological defense and responding to immunotherapy. In addition, patients with low risk were more likely to benefit from ICI therapy than patients with high risk because the subgroup with low risk had lower TIDE scores than those with high risk (Figure 7G). Patients with low risk may have a better prognosis than patients with high risk for patients with lower TIDE scores. Additionally, we discovered that the two risk subgroups differed significantly in terms of the T cell exclusion score (Figure 7I) and microsatellite instability (MSI) score (Figure 7H), except T cell dysfunction (Figure 7J). Compared to TIS and TIDE, the AUC result showed that our risk model was better (Figure 7K). Our AUC analyses of NETs-score showed improving accuracy for OS rates at 1, 3, and 5 years (Figure 7L).

**Figure 8:**
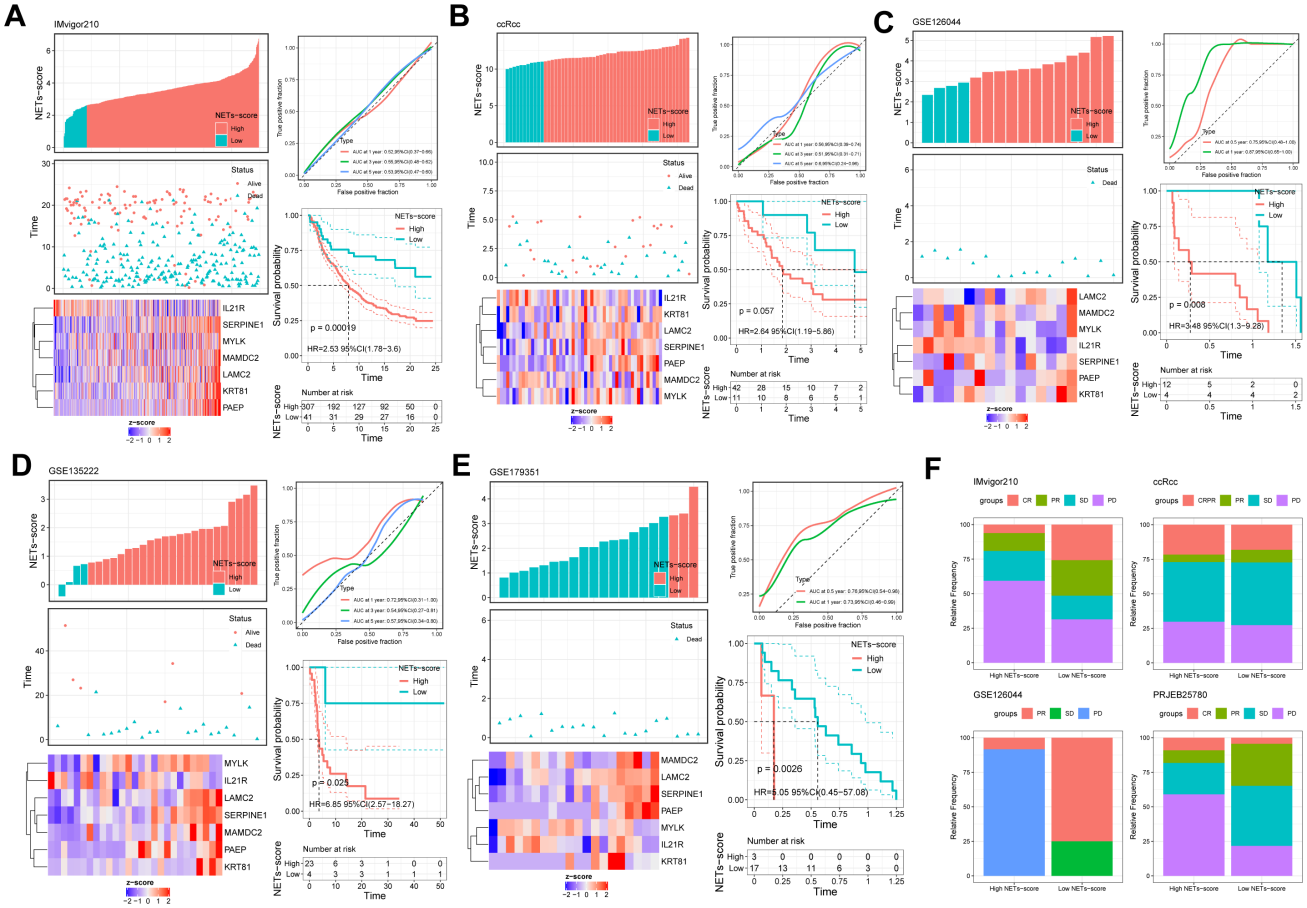
Prognostic value of NETs-scores in different external immunotherapy cohorts (KM survival analysis based on the best cut-off of NETs-score). The upper left parts are distribution plots for the relationship between NETs-score and survival status; the lower left parts are heat-maps for the seven prognostic NETs-related genes in the cohorts; the upper right parts are ROC curve for the NETs-score in the different internal and external cohorts; the lower right parts are survival curves between high- and low-NETs-score groups. **(A)** IMvigor210; **(B)** ccRcc; **(C)** GSE126044; **(D)** GSE135222; **(E)** GSE179351; **(F)** The relative frequency of different clinical responses among patients with a high or low NETs-score in different immunotherapy cohorts.

The anti-PDL1 immunotherapy cohort (IMvigor210) allowed us to further test this idea, and we found that patients in the low-RS score group had a longer median survival time and a better prognosis than those in the high-RS score group (Figure 8A). It was shown that patients with Complete Response (CR) and Partial Response (PR) were more prevalent in the low-RS score group, whereas those with Stable Disease (SD) and Progressive Disease (PD) were more prevalent in the high-RS score group (Figure 8F). Consistency with the finding of IMvigor210, the other external immunotherapy cohort showed that patients in the low-NETs score group had a longer median survival time and a better prognosis than those in the high-NETs score group (Figures 8B–E). Moreover, we use the NETs-score to correlate the relevant immune indicators in pan-cancer (Supplementary Figure S7). Focusing on stomach cancer, the NETs-scores is strongly related to relevant immune indicators.

### Drug sensitivity

**Figure 9:**
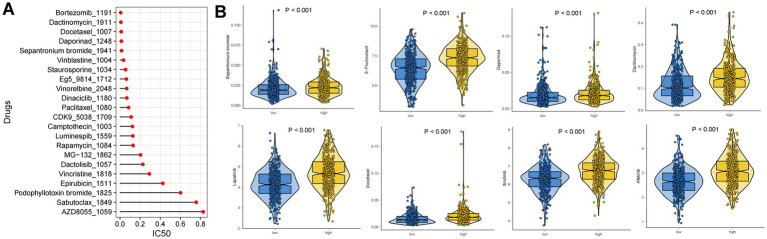
Drug sensitivity analysis. **(A)** IC50 testing results for drugs with IC50 < 1 in different NETs-score groups. **(B)** Potential drugs with significant treatment differences in the different NETs-score groups.

Ten possible anticancer medications with IC50 < 1, suggesting a significant inhibitory impact on GC, were found (Figure 9A). In different risk groups, there were statistically significant variations in how each drug responded (Figure 9B). We illustrated that a low risk was associated with a lower half inhibitory concentration (IC50) of chemo-therapeutics such as Dactinomycin, Afatinib, Daporinad, Ibrutinib, Docetaxel, Lapatinib, Sepantronium bromide and 5-Fluorouracil (*p* < 0.05). Therefore, Figure 9 illustrates that the NETs-Score acted as a potential predictor for chemosensitivity.

### The pan-cancer analysis of NETs-score

To assess the similarities and differences of the risk score model between various malignancies, we conducted a pan-cancer analysis. We thoroughly examined TMB, MSI, and CD274 expression in various malignancies (Supplementary Figure S6). In THYM, LGG, and SARC, the NETs-score was favorably connected with TMB (*p* < 0.05), but in BRCA, LIHC, BLCA, UCEC, PRAD, LUSC, and STAD, the correlation was inversely correlated with TMB (*p* < 0.05). A relationship between MSI and TGCT, UVM, as well as STAD, CHOL, and ESCA, was shown to be both positive and negative. Additionally, the NETs-score was inversely connected with CD274 content in LAML, HNSC, THCA, KIRC, SKCM, STAD, BRCA, TGCT, and LUSC but positively correlated with CD274 expression in GBM, ACC, LGG, BRCA, LIHC, PAAD, ESCA, BLCA, and PRAD. We also determined the connection between the NET score and 22 indices of immune cell infiltration and stemness. Supplementary Figure S6 presents the comprehensive outcome.

### Immunohistochemistry staining (IHC) for the signature genes

**Figure 10:**
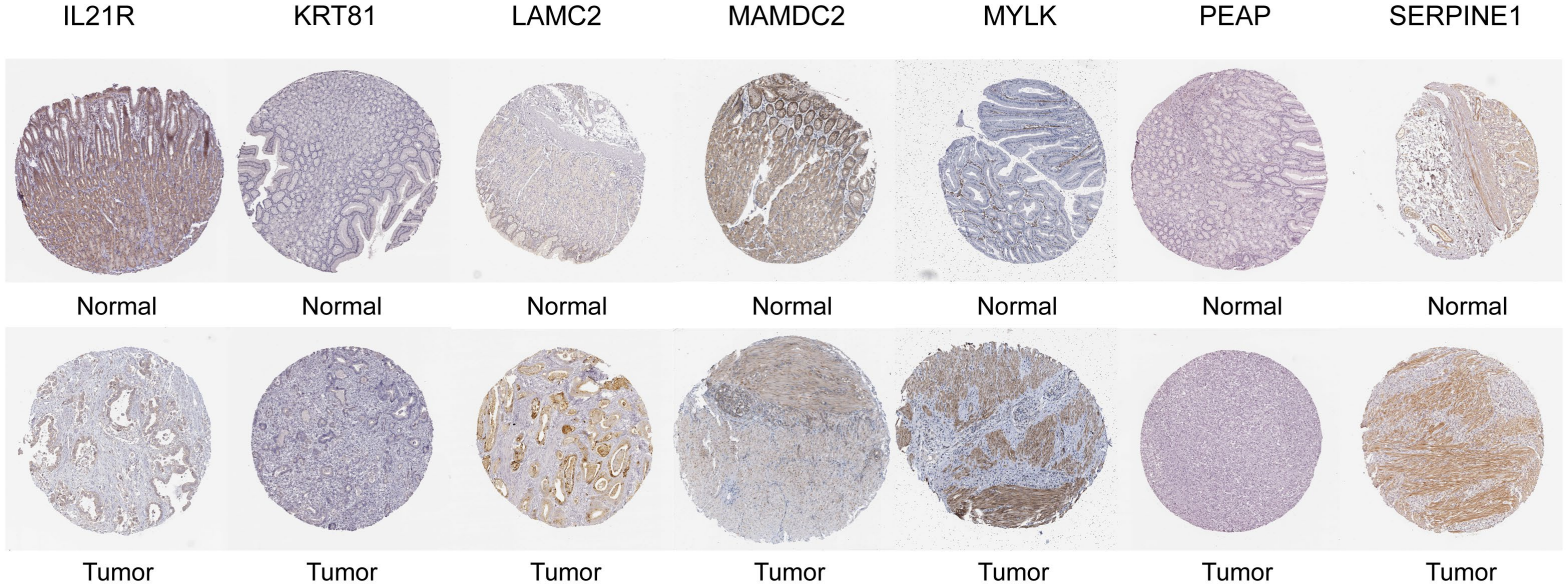
Representative immunohistochemical staining images of SERPINEI (antibody CAB068501, 10x), LAMC2 (antibody CAB078165, 10x), MYLK (antibody CAB020789, 10x), MAMDC2 (antibody HPA021814, 10x), PAEP (antibody HPA020108, 10x), IL21R (antibody HPA042296, 10x), and KRT81 (antibody HPA049778, 10x) in normal and STAD tissues are retrieved from The Human Protein Atlas database (HPA, https://www.proteinatlas.org/, accession date: June 2023).

We have analyzed the protein expression profiles derived by immunohistochemical staining, which are accessible in the HPA database, in order to further confirm the expression patterns of the signature genes in GC patients. The results indicated that seven risk genes (SERPINE1, LAMC2, MYLK, IL21R, KRT81, MAMDC2, and PAEP) of the signature were differentially expressed in the cancer tissues compared to the normal tissues (Figure 10).

## Discussion

The third highest case fatality rate of all cancers, gastric cancer (GC), is the fifth most common malignancy worldwide (1). The main therapies for GC are still surgical excision, radiation, and chemotherapy, although the prognosis is still poor (2). Clinical, epidemiological, and translational studies are mainly focused on GC, which is still a frequent cancer (36). To capture microorganisms, neutrophils produce neutrophil extracellular traps (NETs), which are made of chromatin DNA filaments wrapped with granule proteins (37–39). A transmembrane DNA receptor that mediates NET-dependent metastasis was identified by Yang L’s study (40). This demonstrated that the NETs are made up of externalized DNA of mitochondrial or nuclear origin that is coupled to histones and granular proteases such as neutrophil elastase (NE) and myeloperoxidase (MPO) (41). Given that NETs catch microorganisms, it has been hypothesized that their pro-metastatic effects result from the capture of cancer cells that have spread throughout the body (42), but the specifics of this interaction between NETs and cancer cells are yet unknown. Consequently, this study offers fresh perspectives for enhancing the prognosis of GC patients and advancing our understanding of the precise molecular pathways of NETs for GC.

First, we pulled 353 NET-related genes from the GEO database, GSE188741. 27 NETs-related genes were identified by univariate Cox regression analysis from TCGA plus GSE84437 (*p* < 0.05) based on these NETs-related genes. A high amount of mutations are present in CUBN and FLNC. Some literature found that the CUBN and FLNC were associated with altered gastric cancer risk and involved the lymph node metastasis of gastric carcinoma, respectively (43, 44). With the help of consensus clustering algorithms, it is possible to quickly examine and locate groups of patients with various features in a vast quantity of data (45). Therefore, based on the levels of expression of 27 NETs-related genes, we employed this unsupervised algorithm to distinguish between two different molecular subtypes. We discovered that patients with subtype A had a higher chance of surviving than those with subtype B. In order to explore the differences in biological behavior between these two subtypes, we also performed a GSVA enrichment analysis. According to some published research, neutrophil extracellular traps (NETs) are extracellular strands of decondensed DNA in association with histones and granule proteins that are released by dying neutrophils in order to catch and kill bacteria (46). NETs could be more significant in thrombosis and autoimmunity (41). The clinical prognosis of gastric cancer was independently predicted by focal adhesion-related proteins (47). In consistence with the finding, subtype B has pathways that were particularly linked to focal adhesion, dilated cardiomyopathy, cell cycle, DNA replication, and calcium signaling.

In this work, we created an incredibly potent prognostic NETs score and showed how it might predict outcomes. The NETs score is calculated using the expression levels of seven genes (SERPINE1, LAMC2, MYLK, IL21R, KRT81, MAMDC2, and PAEP) and used to GC patients to demonstrate its prognostic power. In order to evaluate the similarities and differences of the risk score model among other malignancies, we also performed a pan-cancer study. The outcome demonstrated how powerful a predictive model the risk score was. In gastric cancer cell lines and tissues, LAMC2 gene expression and DNA methylation analysis revealed that DNA hypomethylation was linked to LAMC2 up-regulation (48). These findings imply that LAMC2 activation may be crucial to the development of gastric cancer. Numerous studies have shown that SERPINE1 overexpression is associated with tumor development and bad outcomes in a variety of malignancies, including GC (49, 50). Poor prognosis is linked to SERPINE1’s aberrant expression, which has been seen in many different cancer types. In particular, SERPINE1’s function in tumor angiogenesis has been well studied. Its effects are achieved through the control of endothelial cell plasmin-mediated proteolysis (51), migration (52, 53), and apoptosis (54). The immunological control of immune cells and the development of tumors are regulated by the interleukin-21 receptor (IL-21R) in a variety of malignancies. By attaching to its receptor, IL-21R, interleukin (IL)-21, a member of the IL-2 family, is implicated in biological processes in cancer and autoimmune disease (55). According to research, the expression of MYLK was greater in GC tissues than in nearby normal tissues (56). Furthermore, transcription factors, including AR-v12 and methylation, were both used to control MYLK (56). The other signature genes are represented by immunohistochemistry staining (IHC) according to The Human Protein Atlas database, which shows the different expressions in cancer tissue compared with the normal. However, these genes are still proven in further research.

We created and verified a nomogram by merging NET score, age, and pathological stage in order to further increase the accuracy of prognostic prediction. The outcome demonstrated that the prognosis of GC was highly correlated with age, risk score, and clinical stage. As we know, the prognosis of cancer is strongly associated with the pathological stage of pan-cancer. We determined the NET score provided as an independent prognostic factor in the context of the results.

Higher TMB produces more neo-antigens, which improves the likelihood that T cells will recognize them and clinically corresponds with higher ICI results (57). Therefore, patients with elevated TMB often have favorable survival rates, according to several research on different tumors (58). In consistence, we showed that increased TMB was seen in the low-risk subgroup of the NETs-score. This indicates that patients with high TMB had considerably better OS than those with low TMB. In this investigation, we discovered that the most prevalent mutant genes for both groupings were TTN, TP53, and MUC16. These gene mutations have been used in certain literature to predict outcomes and guide immunotherapy (58–62). A gene combination, MUC4, MUC16, and TTN, was recently proposed by Yang et al. to predict TMB, and this gene combination may serve as a more practical and affordable biomarker for immunotherapy effectiveness in place of TMB (60). Patients with a high degree of microsatellite instability (MSI-H) respond to immunotherapy better and may benefit from it, as is well known (63). Therefore, the immunotherapy was more beneficial for the GC patient with a low NET score. Furthermore, GC cells with a lower NET score had more evident stem cell traits and a lower level of cell differentiation.

To assess the importance of immune cell infiltration in GC with various risk categories for our inquiry, the relative number of 22 immune cells in each GC specimen was analyzed using CIBERSORT. The two main types of monocytes detected in the blood are macrophages M1 and M2, as is common knowledge. M2 macrophages may also contribute to the growth of GC tumors, according to some research (64, 65). We showed that lower M2 infiltration was linked to a better prognosis, which is well-known that M2 infiltration is a risk factor in pan-cancer. According to the literature, infiltrating mast cells are frequently seen in GC, which is associated with tumor development and indicates a poorer OS (66). We demonstrated that a worse prognosis was associated with more mast cell infiltration, which is consistent with this study. In murine models, CD4+ neoantigen-specific T cells found inside tumors are necessary for the immune system to respond to immune checkpoint blockage (67). These cells may also facilitate tumor rejection by directly destroying tumor cells (68), inducing the body’s innate immune system (69), and stimulating CD8+ T cells (70). According to published research, neutrophils build up in the peripheral blood of cancer patients, particularly in those with advanced-stage illness, and a high circulating neutrophil-to-lymphocyte ratio is a reliable indicator of a poor clinical prognosis in a variety of malignancies (71). The evidence led us to believe that the NETs-Score could represent immune cell infiltration as well as the importance of different immune cell types for prognosis.

In addition, we examined that the low-risk score group had greater expression of various immune checkpoints, such as PD-1 and CTLA-4, as well as the T cell inflamed marker. Moreover, we discovered that the factor of immune subtypes and TCGA subtypes of immune response was highly connected with the risk score. According to the findings, we used IPS to evaluate the NETs-based variations in the TME that could represent various immunological advantages of ICI treatment. The IPS is principally related to a few immunological checkpoints. For the clinical study with immunotherapy, the literature indicated that nivolumab (anti-PD-1) has anti-tumor efficacy and safety in patients with GC, which is delivered as maintenance treatment (after the illness is under control with conventional chemotherapy) (72). In consistence with our results, there were considerably higher in the low-risk group, which was characterized by the NETs-score, predicted the CTLA4-negative-PD1-negative group of IPS. Finally, the NETs-Score was used to group patients from the immunotherapy databases. The median survival time and prognosis were both longer for patients with low RS scores than for those with high RS scores. In addition, the AUC result showed that our risk model was better compared to TIS and TIDE. Moreover, we indicated the NETs-score in stomach cancer is strong relation in relevant immune indicators.

We developed a risk score model to forecast both the outcome of targeted treatment and the outcome of immunotherapy. The clinical research found that immune treatment in GC patients had a fantastic outcome before the disease was controlled by conventional chemotherapy (72). In addition to 5-fluorouracil (5-FU) and platinum, taxanes have also shown action in GC patients, with Docetaxel and Paclitaxel both demonstrating a survival benefit in first-line and second-line therapy of patients with metastatic GC, respectively (73, 74). We showed that these medications in the low-risk category had a good possibility for a therapeutic response based on the literature. We also showed that the low-risk group benefits greatly from immunotherapy. It implies that future research can concentrate on the advantages of immunotherapy in conjunction with GC treatment.

Our study attempted to characterize GC patients, identify DEGs and create a predictive model, and relate NETs to patient prognosis. The clinical prognosis, immunological infiltration, and clinicopathological characteristics of GC patients may be recognized using the NETs-score grouping. This study provides knowledge for individualized strategies that will direct immunotherapy and chemotherapy as well as further clarify the role of the NET score in the prognosis prediction value. Even though we verified the study using many databases and angles, there were still certain restrictions that needed to be taken into account. The connections between these model genes and their biological functions deserve more study.

## Data availability statement

The original contributions presented in the study are included in the article/Supplementary material, further inquiries can be directed to the corresponding authors.

## Author contributions

ML, ZZ, and TM conceived and designed this manuscript. ZZ, TM, and XW performed the data analysis and interpreted the data. ML, ZZ, TM, and JC completed the manuscript. HR, Z-WY, and CZ verified the underlying data. All authors contributed to the article and approved the submitted version.

## Funding

This study was supported by the National Natural Science Foundation of China (82073148), Free Exploration Foundation of Science, Technology and Innovation Commission of Shenzhen Municipality (No. JCYJ20180307151238174), Shenzhen Fundamental Research Program (JCYJ20190809142807444) and the Guangdong Basic and Applied Basic Research Foundation (2020A1515110303).

## Conflict of interest

The authors declare that the research was conducted in the absence of any commercial or financial relationships that could be construed as a potential conflict of interest.

## Publisher’s note

All claims expressed in this article are solely those of the authors and do not necessarily represent those of their affiliated organizations, or those of the publisher, the editors and the reviewers. Any product that may be evaluated in this article, or claim that may be made by its manufacturer, is not guaranteed or endorsed by the publisher.

## Footnotes

^1^ https://portal.gdc.cancer.gov/

^2^ https://www.ncbi.nlm.nih.gov/geo/

^3^ https://cibersort.stanford.edu/

^4^ HTTP://tide.dfci.harvard.edu/

^5^ https://www.cancerrxgene.org/
