# Supplementary figures and images for: Neutrophil extracellular traps−related signature predicts the prognosis and immune infiltration in gastric cancer
Source: Front Med (Lausanne). 2023 Aug 10;10:1174764. doi: 10.3389/fmed.2023.1174764 (PMC10447905; doi:10.3389/fmed.2023.1174764)

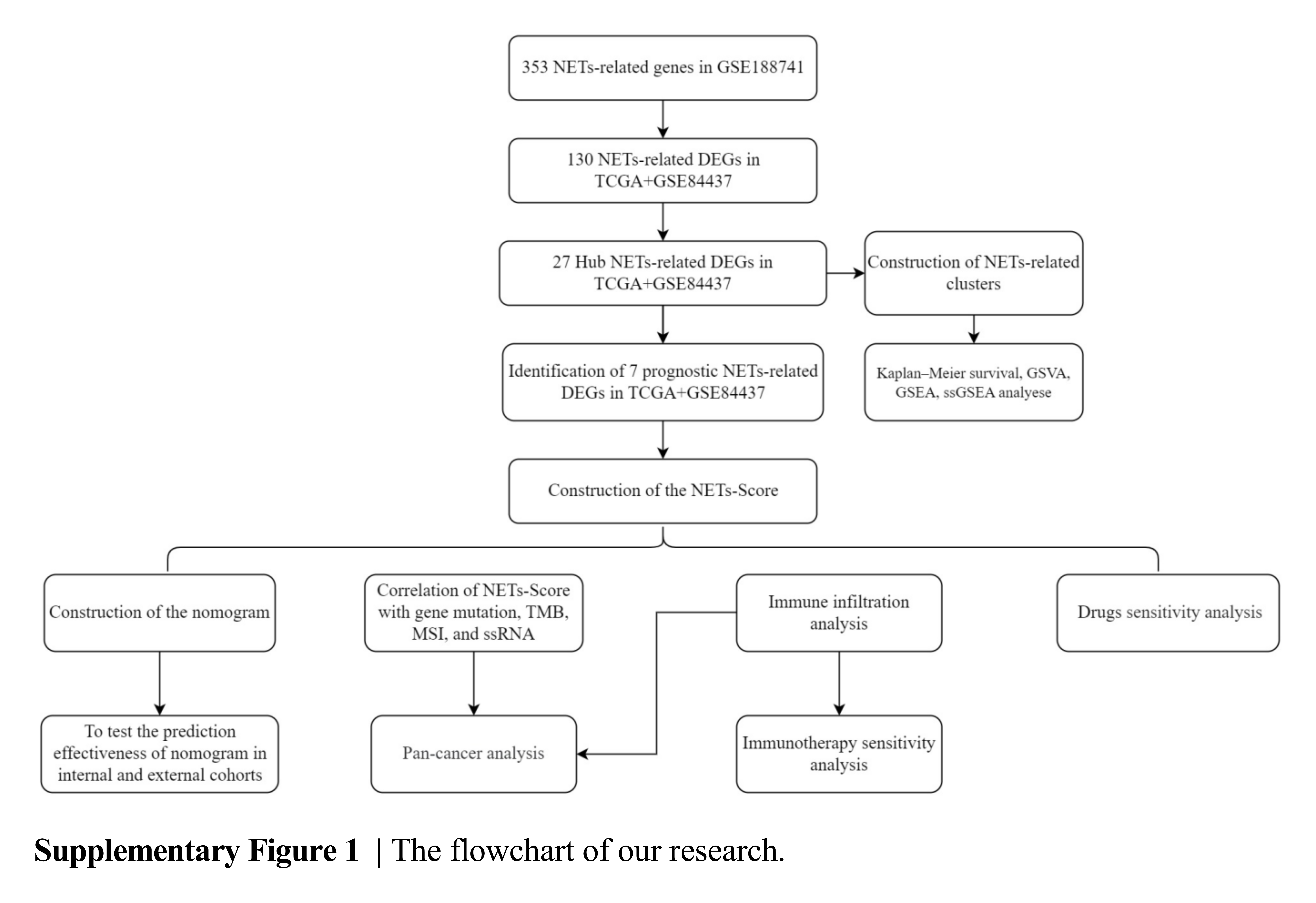

Supplement: Supplementary file 1 [file Image_1.TIF]

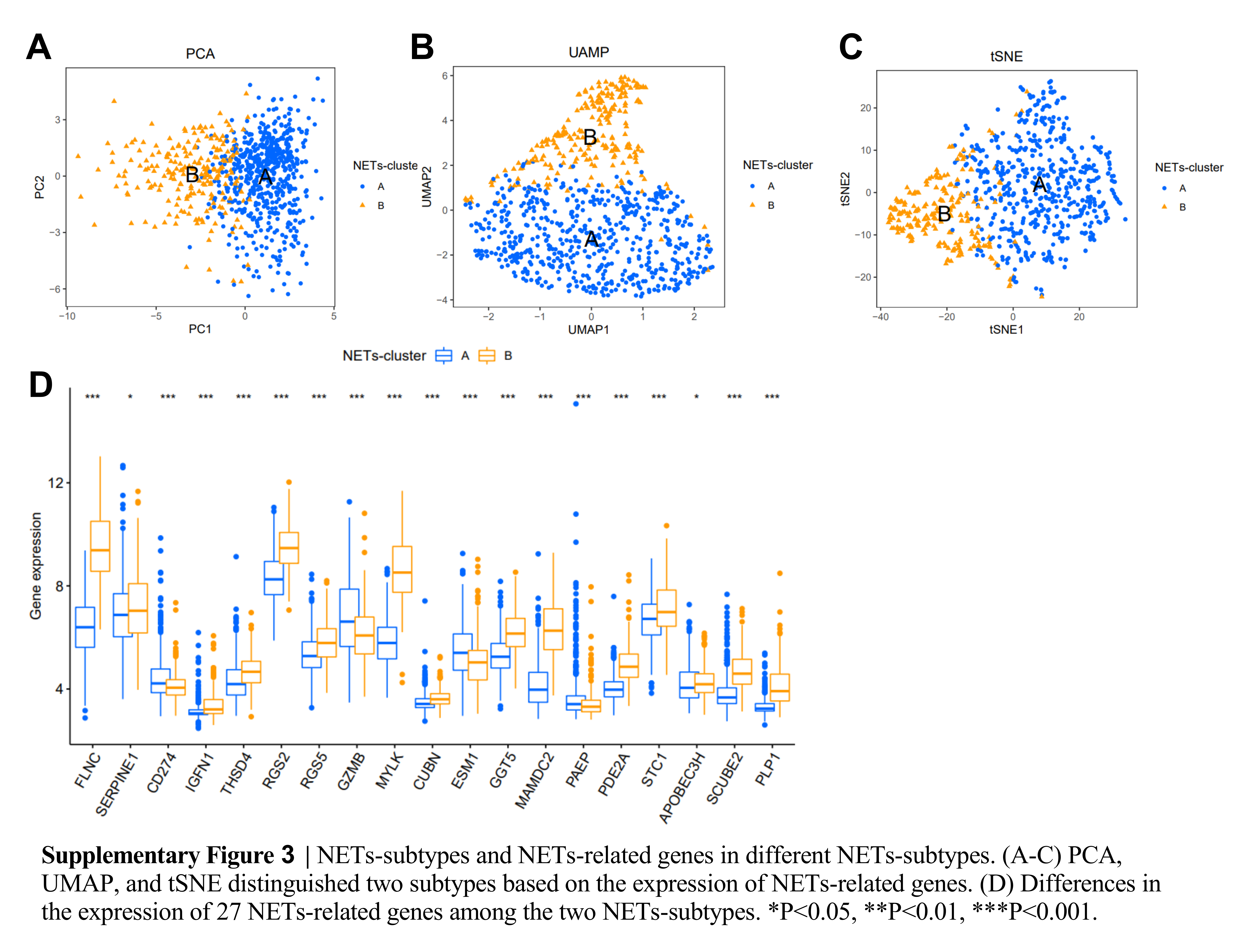

Supplement: Supplementary file 3 [file Image_3.TIF]

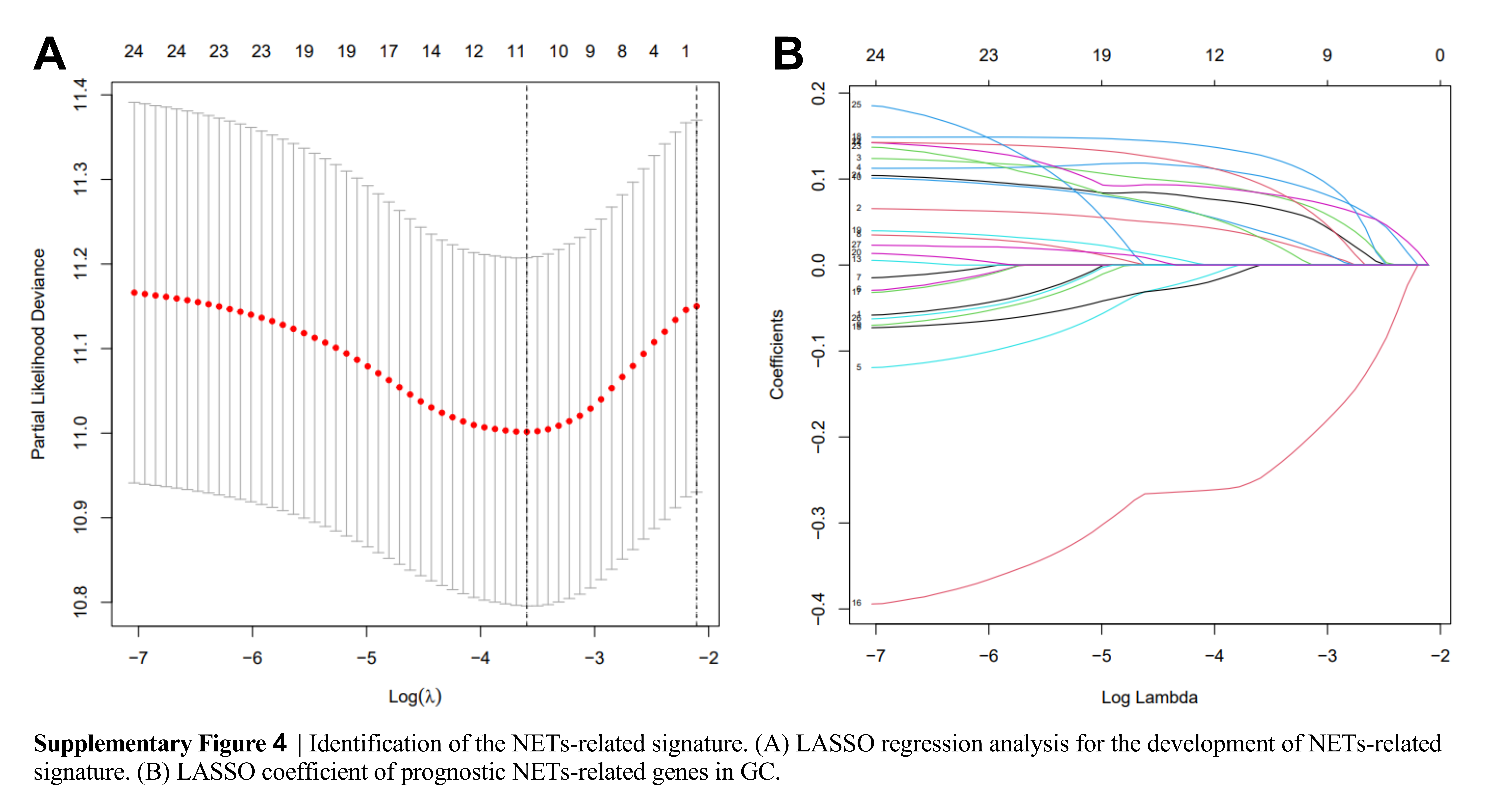

Supplement: Supplementary file 4 [file Image_4.TIF]

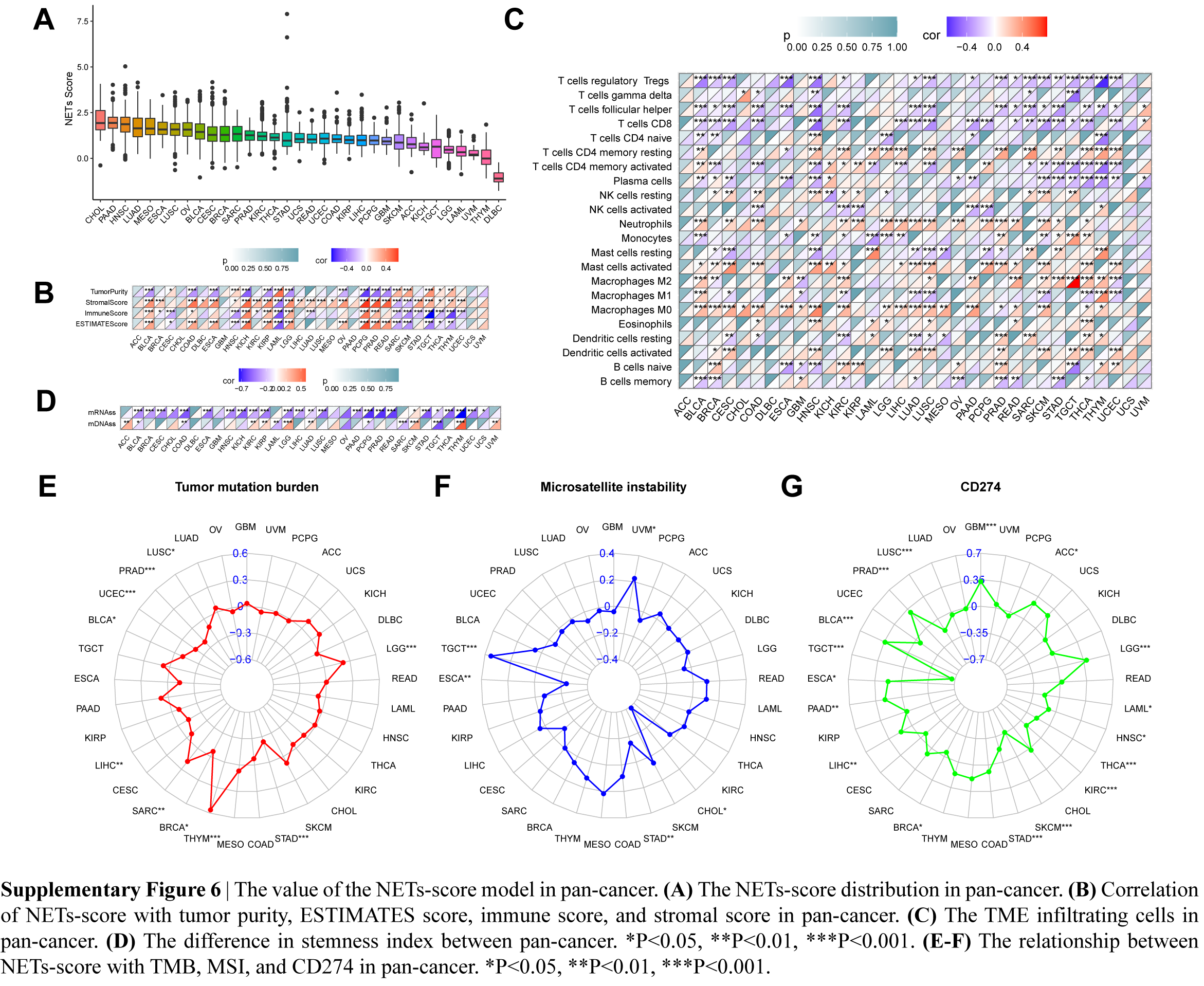

Supplement: Supplementary file 6 [file Image_6.TIF]

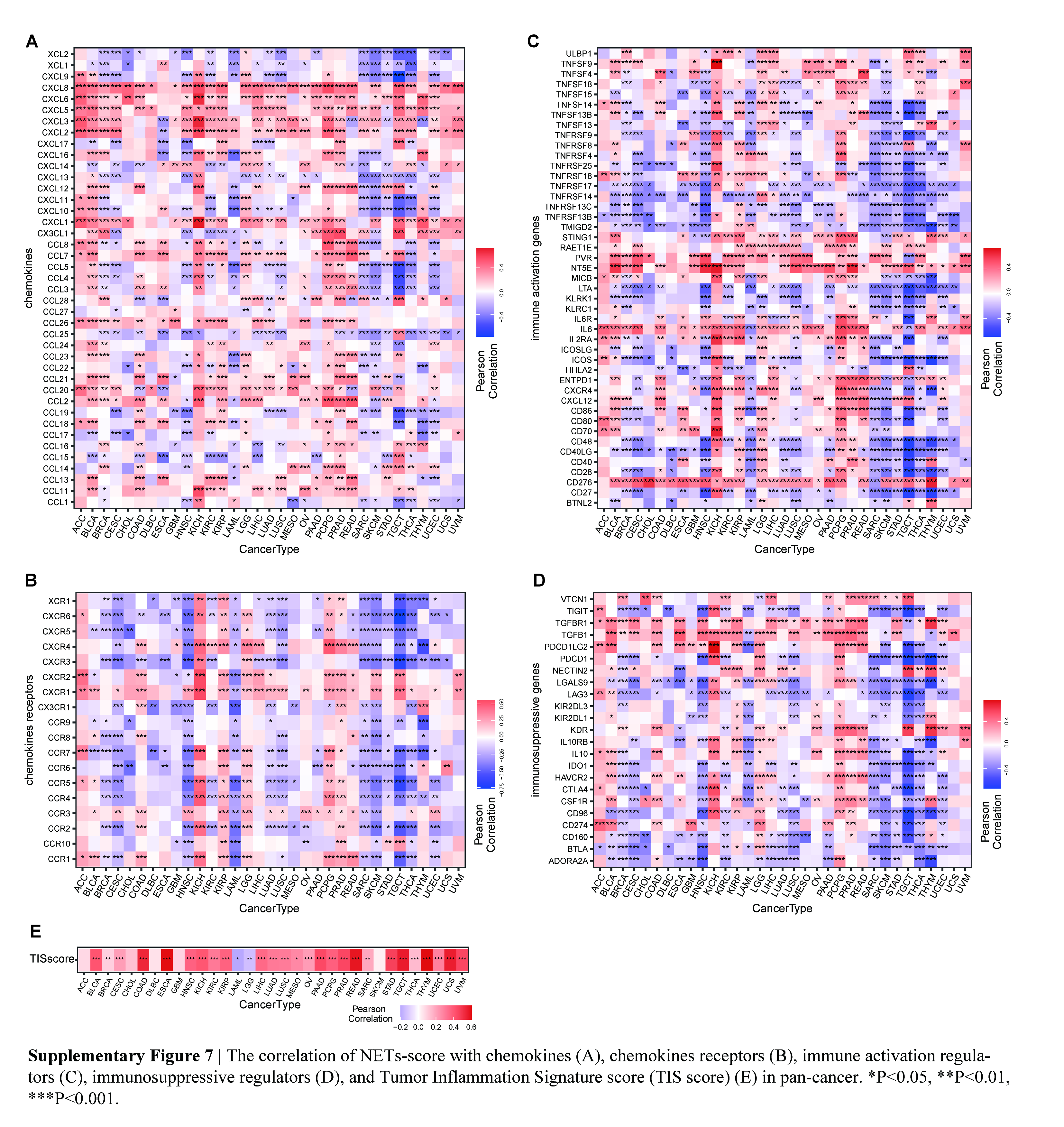

Supplement: Supplementary file 7 [file Image_7.TIF]
